# The effect of a vegan diet with or without resistance exercise on thigh muscle volume in older adults. Research protocol of the Vold-study: a 12-week randomized controlled trial

**DOI:** 10.1186/s12877-025-06708-9

**Published:** 2025-12-26

**Authors:** Jacintha Domić, Pol Grootswagers, Philippe JM Pinckaers, Inge Groenendijk, Josep Rubert, Luc JC van Loon, Lisette CPGM de Groot

**Affiliations:** 1 Division of Human Nutrition and Health, Wageningen University & Research, P.O. Box 17, Wageningen, 6700 AA The Netherlands; 2 Department of Human Biology, Institute of Nutrition and Translational Research in Metabolism (NUTRIM), Maastricht University Medical Centre+, Maastricht, The Netherlands

**Keywords:** Plant-based food, Animal-based food, Skeletal muscle, Muscle mass, Aging, Sustainable food

## Abstract

**Background:**

Plant-based diets are increasingly adopted. Plant-based foods exhibit a lower protein quantity and quality compared to animal-based foods. As such, a fully plant-based, i.e. vegan, diet may be suboptimal for the maintenance of skeletal muscle mass later in life. The primary objectives of this study protocol are therefore: (1) To assess the effect of a 12-week self-composed vegan diet in comparison to an omnivorous diet on thigh muscle volume in community-dwelling older adults; and (2) To assess the effect of a 12-week self-composed vegan diet combined with twice-weekly resistance exercise (RE) on thigh muscle volume in comparison to a vegan diet without RE in community-dwelling older adults.

**Methods:**

Seventy-two community-dwelling individuals aged ≥ 65 years with a BMI between 23 and 32 kg/m^2^ will be included in this randomized controlled trial. Eligible participants will be randomly allocated to either follow their habitual omnivorous diet, a self-composed vegan diet, or a self-composed vegan diet combined with two sessions of RE per week for 12 weeks. Participants will be guided through monthly nutrition information meetings. Dietary intake and physical activity levels will be assessed using food records and accelerometery. The primary study outcome will be thigh muscle volume, assessed at baseline and after 12 weeks using magnetic resonance imaging (MRI). Secondary outcomes will be: body composition, muscle fat infiltration, muscle strength, bone mineral density, bone turnover markers, metabolic profile, insulin levels, cobalamin, iron and vitamin D status, hs-CRP, gut metabolomics and metagenomics, gastro-intestinal symptoms and dietary intake. Measurements will take place at baseline, after 6 weeks, and after 12 weeks. Additionally, mixed muscle protein synthesis rates will be assessed during the first ten days of the intervention using a deuterium oxide protocol. Data will be analysed using independent t-tests and linear mixed models.

**Discussion:**

The results will provide valuable insights regarding the implications of consuming a vegan diet later in life for skeletal muscle and other health outcomes, and may contribute to the substantiation of the envisaged more plant-based dietary guidelines.

**Trial registration:**

The trial is registered at clinicaltrials.gov (NCT05809466; registered on 22 February 2023).

**Supplementary Information:**

The online version contains supplementary material available at 10.1186/s12877-025-06708-9.

## Introduction

Environmental and health concerns have led to a growing encouragement for consumers in high-income countries to adopt more plant-based diets [1–3]. Plant-based diets are classified based on the proportion and source of animal- and plant-based foods present in the diet. While a vegetarian diet excludes specific animal-based food groups, as meat and/or fish, a vegan diet represents the strictest form of a plant-based diet and excludes all foods from animal origin. Ample plant-based food consumption holds potential health advantages, including a reduced risk of obesity and cardiometabolic disease [4–8]. However, a strict vegan diet poses a risk for insufficient intake of several nutrients, particularly for protein, vitamin D, cobalamin, calcium, zinc and selenium [9, 10].

An increased risk of deficiency in several of the above-mentioned nutrients typically presents itself later in life as well. The consumption of protein and the status of vitamin D, calcium, and cobalamin have frequently been reported to be low in older individuals. Insufficiencies of these nutrients can lead to adverse effects, including, but not limited to, a reduction in muscle mass, an increased risk of hip fractures and the development of macrocytic anemia, respectively [10–13]. We previously suggested that a vegan diet elevates the risk of an inadequate protein intake in older individuals even further, which may accelerate the age-related decline in muscle mass and function and subsequently increase the risk of sarcopenia [10]. Sarcopenia refers to the age-related decline in muscle strength accompanied by a loss of muscle mass and/or function, typically commencing around the age of 50. It is estimated that up to 30% of older adults residing in community settings experience sarcopenia. Sarcopenic individuals experience an increased susceptibility to falls, hospitalization, and other complications. Adequate dietary protein intake of sufficient quality, particularly in conjunction with resistance exercise (RE), is an established approach in preventing sarcopenia [14].

The ingestion of dietary protein stimulates the growth of muscles through the upregulation of muscle protein synthesis (MPS) and the inhibition of muscle protein breakdown. The degree to which a protein source can promote muscle mass maintenance and accretion predominantly hinges on the quantity and the quality of the protein consumed. Protein quality pertains to the capacity of a protein source to fulfill its functions following digestion and absorption, such as the stimulation of MPS [15]. Plant-based foods are generally considered to have a relatively lower protein quantity and quality in comparison to animal-based foods. Plant-based foods frequently exhibit an incomplete amino acid profile. Additionally, the concurrent abundance of anti-nutritional factors and dietary fibers in plant-based foods further lowers their protein digestibility, and as such, the bioavailability of the essential amino acids within the protein [16, 17]. The lower protein functionality of a protein source that results from its low protein quality is particularly important for older individuals, considering the already blunted MPS response to protein intake typically seen later in life (i.e. anabolic resistance) [18, 19]. Illustrating this, the consumption of 30 g of wheat protein hydrolysate has been shown to increase MPS rates in young individuals, while a similar amount did not increase MPS in older individuals [20, 21].

We previously observed a substantial and significant difference in the post-prandial MPS response to a vegan meal when compared to an omnivorous meal in older adults [22]. On the other hand, these findings were not confirmed when a highly controlled vegan diet providing ample protein was consumed for several days by older adults in a recent study from our group [23], and in another study [24]. Although providing a high internal validity, these results do not reflect the impact a longer term, self-composed vegan die may have, as food, and as such nutrient, intake will be largely different. Furthermore, it remains largely unclear to what extent diet induced changes in mixed MPS affect the longer-term adaptations in muscle mass [25]. Both the acute and longer-term effects of consuming a *self-composed* vegan diet on muscle- and other health related outcomes in the later stages of life have yet to be fully understood.

Considering the high environmental impact of animal-based food production, and the concurrent demand for high-quality protein consumption later in life, it is urgently needed to assess the effects of a vegan diet on skeletal muscle and other health related outcomes in older adults. Thereby shifting from a highly controlled setting and testing isolated proteins and controlled diets [20, 22–24, 26], towards longer-term adaptations to a self-composed vegan diet. Additionally, strategies that may aid muscle maintenance in older individuals while consuming a vegan diet will have to be explored. Therefore, the current paper describes a protocol for a randomized controlled intervention trial with three parallel arms that aims to assess the effect of a self-composed 12-week vegan diet on skeletal muscle mass, strength, and other health related outcomes in comparison to (1) an omnivorous diet, and (2) in comparison to a self-composed vegan diet with concomitant RE, in community-dwelling older adults. We hypothesize that switching to a self-composed vegan diet for 12 weeks leads to a small decrease in thigh muscle volume in comparison to an omnivorous diet, and that concurrent RE will attenuate these losses. An overview of all study objectives is presented in Table 1.

**Table 1:** Primary, secondary and explorative objectives of the Vold-study

**Primary objectives** •To assess the effect of a 12-week self-composed vegan diet in comparison to an omnivorous diet on thigh muscle volume in community-dwelling older adults. •To assess the effect of a 12-week self-composed vegan diet combined with twice-weekly RE on thigh muscle volume in comparison to a self-composedvegan diet without RE in community-dwelling older adults. **Secondary objectives** •To assess the effect of a 12-week self-composed vegan diet in comparison to an omnivorous diet in community-dwelling older adults on: -Other body composition components (fat distribution; (appendicular) lean mass); -Muscle strength; -Bone mineral density and bone turnover markers; -Metabolic profile; -Insulin levels; -Vitamin B12, vitamin D and iron status; -hs-CRP; -Gastro-intestinal symptoms; -Gut metabolomics and metagenomics. •To assess the effect of a 12-week self-composed vegan diet combined with twice-weekly RE in comparison to a self-composed vegan diet without RE in community-dwelling adults on: -Other body composition components (fat distribution; (appendicular) lean mass); -Muscle strength; -Bone mineral density and bone turnover markers; -Metabolic profile; -Insulin levels; -Vitamin B12, vitamin D and iron status; -hs-CRP; -Gastro-intestinal symptoms. •To assess the effect of a *10-day* self-composed vegan diet in comparison to an omnivorous diet on mixed MPS rates in community-dwelling older adults. •To assess the effect of a *10-day* self-composed vegan diet combined with twice-weekly RE in comparison to a self-composed vegan diet without RE on mixed MPS rates in community-dwelling older adults. •To assess the nutritional composition of a self-composed vegan diet consumed by community-dwelling older adults. **Explorative objectives** •To assess the effect of a 12-week self-composed vegan diet in comparison to an omnivorous diet, and in comparison to a self-composed vegan diet combined with twice-weekly RE in community dwelling older adults on: -Body mass; -Immune markers; -(Osteo)arthritis complaints in (osteo)arthritis patients; -DNA methylation.	

Abbreviations used: *RE* Resistance exercise, *MPS* Muscle protein synthesis, *hs-CRP* High sensitive c-reactive protein

## Methods and design

### Study design

#### Study overview

**Fig. 1:**
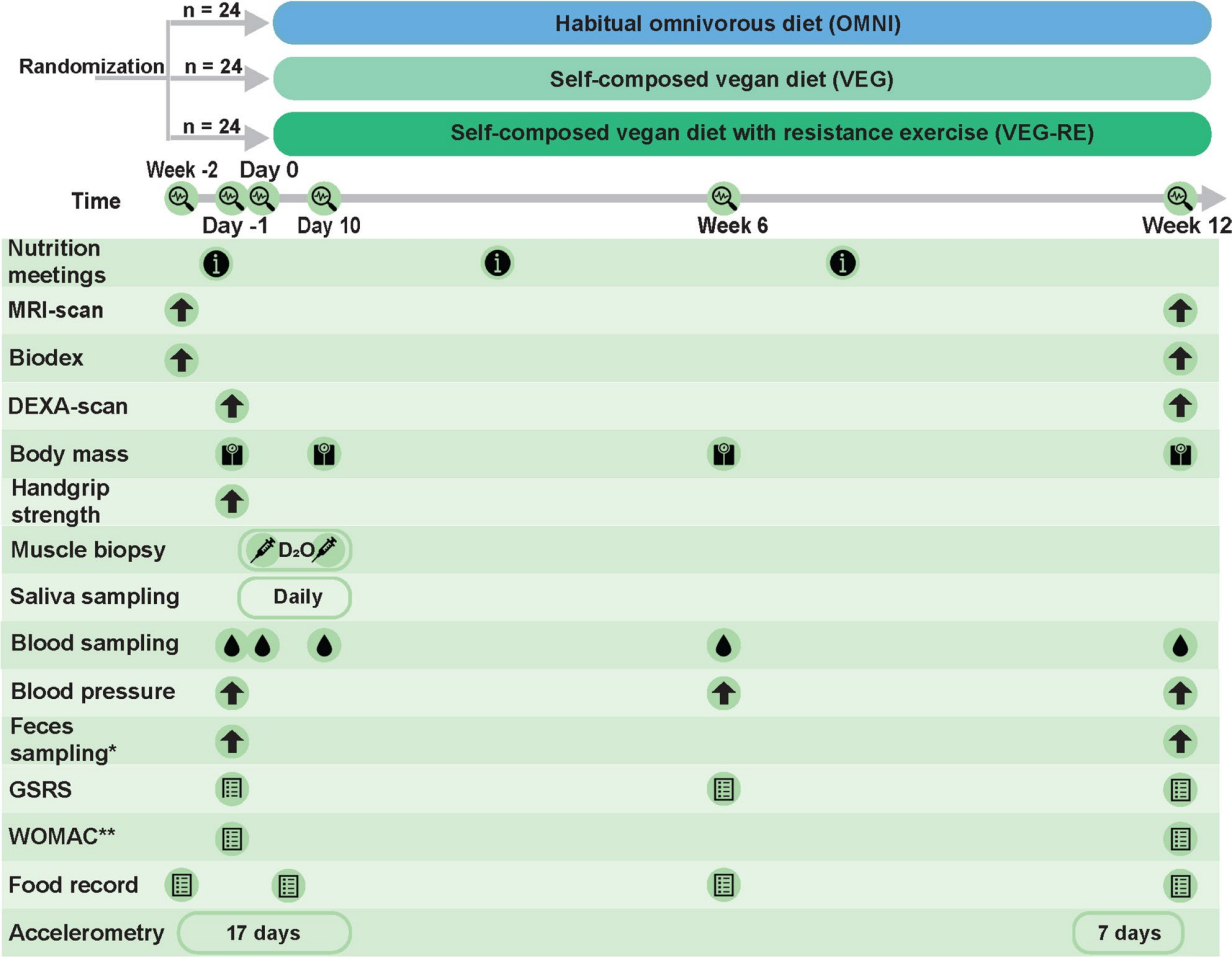
Study design. *Not in the VEG-RE-group. ** only in participants diagnosed with (osteo) arthritis at the moment of screening. Abbreviations: OMNI, the participants allocated to consume their habitual omnivorous diets; VEG, the participants allocated to consume a vegan diet; VEG-RE, the participants allocated to consume a vegan diet and participate in a resistance exercise program; MRI, magnetic resonance imaging; DEXA, Dual X-Ray Absorptiometry; D_2_O, deuterium oxide; GSRS, gastro-intestinal symptom rating scale; WOMAC, Western Ontario and McMaster Universities Osteoarthritis Index.

This study is an open-label, single-center, randomized controlled trial with three parallel study arms. A general overview of the design is presented in Fig. 1. The study duration for enrolled participants will be approximately 14 weeks. Following inclusion, the participants will be randomized to follow either their habitual omnivorous diet (OMNI), a self-composed vegan diet (VEG), or a self-composed vegan diet with concurrent resistant exercise twice a week (VEG-RE) for 12 weeks. All groups will be guided through monthly nutrition information meetings. Measurements to assess the study outcomes will be performed at baseline, after 10 days, after 6 weeks and after 12 weeks. Additionally, all participants will follow a deuterium oxide protocol during the first ten days of the intervention period to allow for the assessment of mixed MPS rates. A detailed description of all measurements can be found in the subsequent paragraphs. Measurements will take place at the hospital Gelderse Vallei (Ede, The Netherlands) and at the Human Nutrition Research Unit at Wageningen University and Research (Wageningen, The Netherlands). All study measurements will be performed according to strict standard operating procedures. The duration the study visits range from one to three hours. The study will be conducted in accordance with the Declaration of Helsinki and has been approved by the medical ethics committee Oost-Nederland (METC-Oost; NL82788.091.22). The trial is registered at clinicaltrials.gov (NCT05809466) and the SPIRIT 2013 checklist related to the manuscript can be found in supplementary Table 1.

#### Sample size calculation

The sample size calculation was based on the primary outcome and was performed in GPower version 3.1.9.4 (Düsseldorf, Germany). Since the current study aims to compare the effect of a vegan diet to an omnivorous diet on thigh muscle volume, and to compare the effect of a vegan diet to a vegan diet in combination with RE on thigh muscle volume (two different contrasts), the sample size calculation was based on testing change in thigh muscle volume between two group comparisons. A separate two-tailed α-level of 0.05 was used for each contrast. A mean change of 4% in thigh muscle volume was expected in the vegan group. The latter was based on previous studies that assessed changes in thigh muscle volume during caloric restriction in middle aged to older adults, as no study has previously assessed the effect of a vegan diet on thigh muscle volume. Reductions in thigh muscle volume of ~ 6.5% were previously observed following a year of caloric restriction [27, 28]. Others additionally observed that the largest decline in thigh muscle volume presented itself during the first three months of caloric restriction [29]. Additionally assuming an expected reduction in the consumption of protein with a concurrent reduction in protein quality [10, 30], a mean change of 4% in thigh muscle volume was expected on the vegan diet in the present study. Using an expected mean change of 4% and standard deviation of 5% [10, 27–30], and 80% power, a total of 60 participants, 20 per group, will need to be included in the study. Considering a drop-out rate of 20%, 24 participants per group will be recruited, leading to a total of 72 participants.

#### Participants

**Fig. 2:**
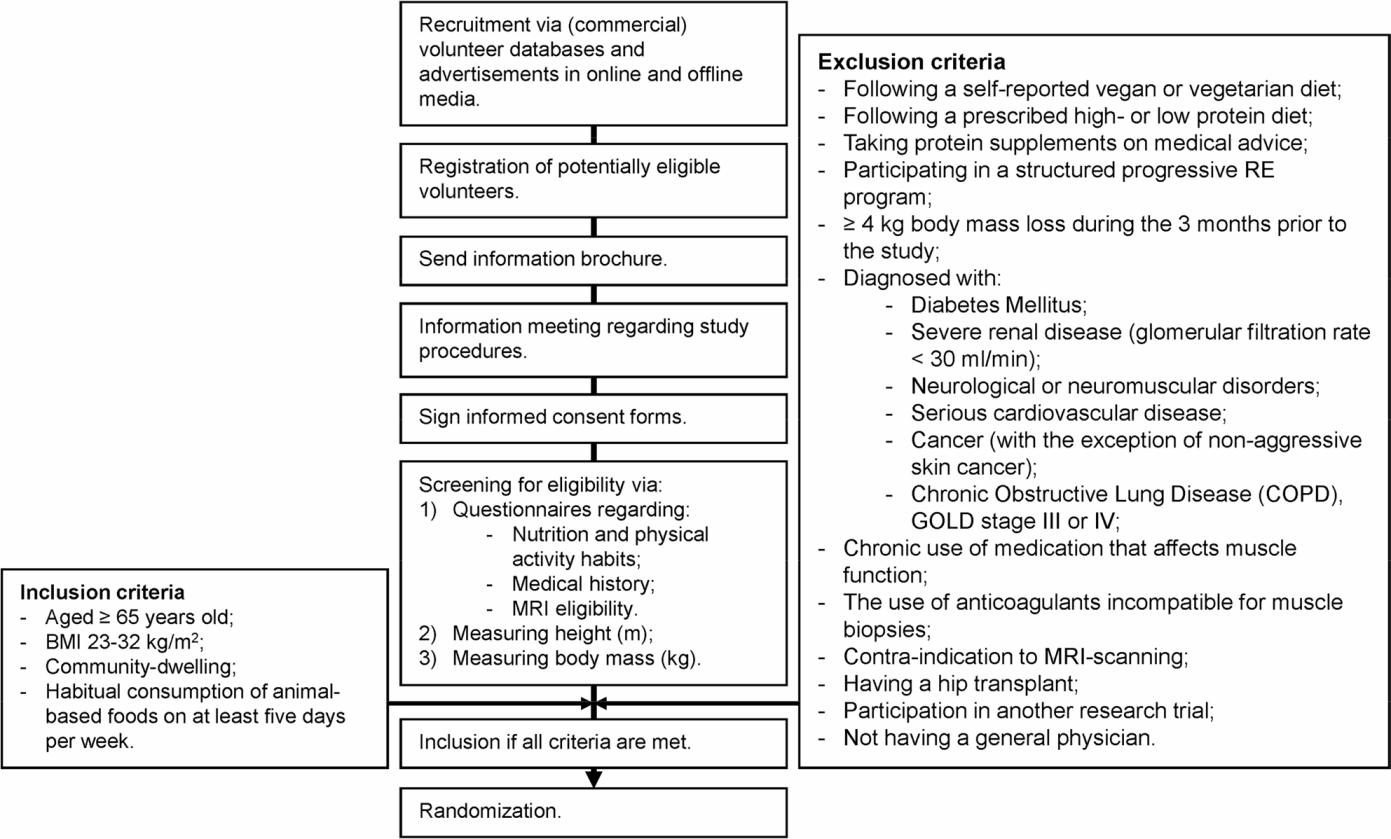
Flowchart of the recruitment process. MRI, magnetic resonance imaging; RE, resistance exercise; BMI, body mass index

Older individuals will be recruited via a volunteer database of the university, posters in supermarkets and other communal locations, advertisements in online and offline media, and commercial volunteer databases. An overview of the recruitment process can be found in Fig. 2. Participants will be considered eligible if they are aged 65 years or older, community-dwelling, exhibit a BMI between 23 and 32 kg/m^2^, and if their habitual diet contains animal-based food products (i.e. dairy, meat or fish) at least 5 days per week. The exclusion criteria include (1) following a self-reported vegetarian or vegan diet during the six months prior to the study, (2) following a prescribed high (≥ 1.2 g per kilogram per day (g/kg/d)) or low protein diet (< 0.8 g/kg/d), and/or or taking protein supplements on medical advice during the month prior to the study, (3) participating in a structured progressive RE training program the during three months prior to the study, (4) having experienced ≥ 4 kg of body mass loss during three months before the start of the study, (5) being diagnosed with diabetes mellitus, severe renal disease (glomerular filtration rate < 30 ml/min), neurological or neuromuscular disorders, serious cardiovascular diseases, cancer (with the exception of the following types of skin cancer: basal cell carcinoma, squamous cell carcinoma), (very) severe chronic obstructive lung disease (COPD; GOLD stage III or IV) or bowel disease, (6) chronic use of medication that affects muscle function, (7) the use of anticoagulants incompatible for muscle biopsies, (8) having a contra-indication to MRI scanning, (9) having a hip transplant, (10) participating in another research trial during the month prior to the start of the study, 11) not having a general physician.

#### Screening

All volunteers will receive detailed written information about the study prior to screening. Additionally, all participants will be informed via a presentation or via telephone prior to screening during which all study procedures will be explained in detail. If still interested, and after providing written informed consent, volunteers will be asked to fill out a questionnaire regarding their demographic information, lifestyle factors and medical history, and a questionnaire regarding MRI contra-indications. The medical history of the volunteers will be checked by the research physician. Additionally, body mass (kg) and height (m) will be measured using a calibrated digital scale and a wall-mounted stadiometer. Participants will be included in the study if they meet all inclusion criteria.

#### Randomization

Following inclusion, participants will be randomized to one of the three study arms on a 1:1:1 ratio, using block randomization with variable block sizes of 3, 6 and 9 to ensure equal allocation to each group. Randomization will be stratified for gender and partners will be randomized together. The randomization will be performed in Castor EDC by a member of the research team [31]. Considering the logistics of the study procedures, both the members of the research team and the participants will be aware of the group allocation.

### Intervention

#### Study diets

All participants will attend monthly nutrition information meetings, commencing in the week prior to the initiation of the study diets. The content presented during these meetings will be based on the Dutch national dietary guidelines [32]. The participants assigned to the VEG and VEG-RE groups will be instructed to abstain from consuming any animal-based food products throughout the intervention period. During the nutrition information meetings, these groups will be informed regarding suitable plant-based replacements for animal-based food products, such as legumes, nuts, and dairy alternatives. Furthermore, these participants will receive guidance regarding the adoption of a vegan diet and will be given the opportunity to address any inquiries or concerns pertaining to the intervention. During these meetings, additional emphasis will be laid on the nutrients protein, cobalamin, vitamin D and calcium. The participants will be equipped with practical resources, including recipes and pocket-size hand-outs containing information regarding, for example, (non)vegan ingredients and vegan product logos.

With respect to the participants assigned to the control (OMNI) group, their habitual dietary intake as omnivores will form the basis for their dietary consumption throughout the intervention period. To address the possibility of potential confounding effects arising solely from increased adherence to the Dutch national dietary guidelines within the vegan groups, the control group will also participate in monthly nutrition information meetings. During these sessions, information about the Dutch national dietary guidelines [32] will be disseminated, including the recommendations regarding the consumption of animal-based food products. The control group will, however, not be strictly instructed to comply with these guidelines.

All participants will be provided with commercially available food products on a bi-weekly basis. Participants can select these products from a predefined list and the provided products will thus vary among participants. The participants in the vegan group will choose from a range of plant-based food products including legumes, nuts, vegetables, and plant-based analogues for dairy or meat, among others. On the other hand, the control group will choose from a predefined list containing only animal-based food products such as meat, dairy, and poultry. By providing these products we aim to assist participants in effectively implementing their assigned diet and to promote adherence across all groups. All participants will further be instructed to discontinue the use of any nutritional supplements one week prior to the intervention, except for those supplements that are medically prescribed and vitamin D supplements, as daily vitamin D supplementation is recommended by the Dutch Ministry of Health for older adults [33]. Participants who were not already taking vitamin D will be advised, but not instructed, to supplement either 10 µg (participants aged < 70) or 20 µg (participants aged ≥ 70) of vitamin D per day. The use of vitamin D supplementation will be recorded.

#### Resistance exercise

Participants assigned to the VEG-RE group will engage in progressive RE twice a week throughout the intervention period. The exercise sessions will be conducted at a separate training facility located in Wageningen. The training protocol employed in this study is based on a pre-existing RE protocol utilized in previous research conducted by our research group [34]. The exercise sessions will have a duration of approximately one hour and will be supervised by an experienced trainer. 

Initially, participants will be introduced to the proper techniques and correct execution of the exercises. Subsequently, sub-maximal strength (8-repetition maximum, 8-RM) of the lower-extremity exercises will be measured to determine the appropriate training load for the exercise protocol. The 8-RM of the lower extremity exercises will be assessed at least every 6 weeks starting at the first training session, and the exercise intensity will be adjusted accordingly. Sub-maximal strength (15-RM) of the upper-extremity exercises will be estimated at the second training session. Each training session will start with a 5-minute warm-up through light exercises. Subsequently, the following exercises will be performed in varying order (1) Leg Press (Body Solid, G9S system, Illinois, USA), (2) Leg Extension (Body Solid, G9S system, Illinois, USA), (3) Lat Pulldown (Body Solid, G9S system, Illinois, USA), (4) Chest Press (Body Solid, G9S system, Illinois, USA), (5) Dumbbell Shoulder Press, and (6) Cable Row (Body Solid, G9S system, Illinois, USA). Due to logistic aspects of the study, participants may perform a Push Up and a Dumbbell Pullover instead of the Chest Press and Lat Pulldown at two of the 24 training occasions during the study. Four sets of 10 to 15 repetitions will be performed for the exercises of the lower extremity, with the intensity ranging from 50% of their estimated 1-RM in week 1 to 75% in week 12. Furthermore, three sets of 15 repetitions will be performed for the upper body exercises, ranging from 50% of their estimated 1-RM in week 1 to 60% in week 12. Finally, all exercise sessions will be concluded with a 5-minute cool-down through light exercises. The experienced trainer will monitor the participants’ progress and well-being regularly.

#### Deuterated water protocol

To determine mixed MPS rates, expressed as daily protein fractional synthesis rates (FSR, %/day), participants will be instructed to follow a deuterated water (D_2_O; or deuterium oxide; purchased from Cambridge Isotope Laboratories and Sigma Aldrich) supplementation protocol for eleven days. The dosing protocol that will be utilized in this study has been previously described elsewhere [35]. The dosing protocol will consist of one dosing day and ten maintenance days. The protocol will be initiated one day prior to the start of the dietary intervention. On this first day, the dosing day, participants will orally consume two doses of 100 mL of 70% enriched D_2_O, with four hours in between the doses. Over the subsequent 10 days, participants will be instructed to consume 20 mL of D_2_O daily to ensure a steady-state body water deuterium enrichment over this period.

#### Compliance

To monitor compliance during the intervention, participants will be provided with a logbook. Participants will be instructed to document any deviations from the study protocol and report any complications related to the dietary changes as well as issues unrelated to the intervention. The logbooks will be reviewed by the research team during each test day, and any issues or concerns identified will be discussed with the participants. Attendance to the RE sessions and nutrition information meetings will be logged. Additionally, the dietary intake of all participant groups will be evaluated at baseline, at the beginning of the intervention, during week 6, and again during week 12 of the intervention, allowing us to assess compliance to the diets after data collection. No feedback will be provided to the participants regarding their dietary intake during the intervention period. Participants will be instructed not to change their habitual physical activity levels during the study, with the exception of the discontinuation of RE. To assess whether compliance to this rule is successful, physical activity will be evaluated at baseline, at the beginning of the intervention and during the last month of the intervention. The procedures are described in more detail in the study outcome section.

### Study outcomes

#### Thigh muscle volume and fat distribution

An overview of all study outcomes is presented in (Table 2). The primary outcome thigh muscle volume (L), and secondary outcomes thigh muscle fat infiltration (MFI; %), liver fat fraction (LFF; %), visceral adipose tissue (VAT; L) and abdominal subcutaneous adipose tissue (ASAT; L), will be assessed using a neck to knee MRI scan (Philips Ingenia Elition 3 T X, Philips Healthcare, Best, the Netherlands) at baseline and after 12 weeks. T1-weighted transverse images will be obtained using a dual-echo FFE scan sequence for the quantification of thigh muscle volume, MFI, VAT, ASAT, and LFF. Participants will enter the scanner head-first. Parameters for the thighs will be: Repetition time (TR), shortest; echo times (TE), shortest; flip angle, 10⁰. Parameters for the abdomen will be: TR, shortest; TE, shortest, 1 ms; flip angle, 10⁰. Image resolution for the scans covering the thighs is 1.84 × 1.84 × 4 mm, and for the scans covering the abdomen is 3 × 3 × 6 mm.

**Table 2:** Overview of the outcome measures

Outcome	Method			Time			
		Screening	Baseline	Day 10		Week 6	Week 12
Primary							
Thigh muscle volume	MRI-scan		X				X
Secondary							
Thigh muscle fat infiltration	MRI-scan		X				X
Fat distribution	MRI-scan; DEXA-scan		X				X
Muscle strength	Biodex		X				X
Mixed muscle FSR	Muscle biopsy		X	X			
	Blood sample		X	X			
	Saliva sample		Daily for 11 days				
Bone mineral density; lean mass	DEXA-scan		X				X
Bone turnover markers	Blood sample		X			X	X
Metabolic profile	Blood sample		X			X	X
Blood pressure	Blood pressure monitor		X			X	X
Micronutrient status	Blood sample		X				X
hs-CRP	Blood sample		X				X
GI health	Stool sample*		X				X
	Blood sample*		X				X
	GSRS		X			X	X
Body mass	Scale	X	X	X		X	X
Dietary intake	Food diary		X	X		X	X
Other							
Participant characteristics	Questionnaire	X					
Height	Stadiometer	X					
WOMAC-index**	Questionnaire		X				X
DNA methylation	Blood sample		X				X
Handgrip strength	Hand dynamometer		X				
Physical activity levels	Accelerometery		Daily for 17 days				Daily for 7 days***
	Activity diary		Daily for 17 days				Daily for 7 days ***
Compliance	Attendance nutrition information meetings	Monthly at each information meeting					
	Attendance RE	Twice a week at each RE session					
	Food diary		X		X	X	X
	Accelerometry		Daily for 17 days				Daily for 7 days***

Abbreviations: *MRI* Magnetic resonance imaging, *DEXA* Dual X-ray Absorptiometry, *GI* Gastro-intestinal, *HS-CRP* High-sensitive C-reactive protein, *GSRS* Gastro-intestinal symptom rating scale, *RE* Resistance exercise, *WOMAC* Western Ontario and McMaster Universities Osteoarthritis Index

^*^Not in the group that is allocated to participate in a resistance exercise program. ^**^only in participants with self-reported (osteo)arthritis at the moment of screening; ^***^ accelerometery may be performed anytime during the last month of the intervention. To be placed on page 23, after the section ‘Other study outcomes’.

Upon arrival after a two hour fast, participants will complete the MRI eligibility form again. Subsequently, participants will change into MRI suitable clothing and will be provided with earplugs and headphones to reduce the scanner noise. A surface coil will be placed on the participants to allow for the measurement of LFF. Communication with the participants will be via the intercom. The participants will be instructed to hold their breath on expiration when the stacks covering the abdomen are obtained to reduce respiratory motion artefacts. The total scanning time for each session will be approximately 13 min. The obtained MRI images will be analyzed using AMRA^®^ Researcher (AMRA Medical, Linköping Sweden), as previously described in detail [36].

#### Muscle strength

Maximal isometric knee extension and flexion strength will be measured using dynamometry (Biodex system V.4X, Biodex Medical Systems, New York). The measurements will be expressed as the maximal voluntary contraction (MVC) in Newton meters (Nm). Prior to the measurement, participants will engage in a 5-minute warm-up on a stationary bicycle. Participants will be seated with their knees flexed at a 60-degree angle and their hips at a 90-degree angle, ensuring stability by securing straps to prevent undesired movements. To familiarize participants with the technique, one submaximal contraction will be performed prior to the measurement. Subsequently, three 5-second MVCs will be executed, with a 30-second rest interval between each contraction. The assessment of knee extension and flexion strength will start with the left leg, followed by measurements of the right leg. There will be a minimum of 2 min rest between the knee extension and flexion measurement. The measurement will be followed by a 5-minute cool down on a stationary bicycle. The highest torques of each leg will be used for analysis.

#### Mixed muscle fractional synthesis rates

Muscle biopsies will be obtained on day 0 and day 10 utilizing the percutaneous needle biopsy technique, as previously described by Bergström et al. [37], with a modified needle (Maastricht Instruments). The biopsies will be obtained from the middle region of the vastus lateralis muscle, 15 cm above the patella and approximately 2 cm below the fascia entry point. In short, the skin and muscle fascia will be locally anesthetized using Lidocaine (20 mg/ml) with Adrenaline (0.01 mg/ml). After approximately 10 min, a small incision of ca. 0.5 cm will be made in both the skin and fascia. Subsequently, the needle will be carefully inserted into the muscle. Using a syringe, a vacuum will be applied to the needle to collect small muscle samples of approximately 50–100 mg. Following the biopsy procedure, the incision will be closed using adhesive strips and covered with a plaster. Furthermore, a pressure bandage will be applied with multiple layers of gauze placed between the incision and the bandage. Subsequently, the muscle biopsies will be dissected carefully and any visible non-muscle material will be removed. The muscle samples will immediately be frozen in liquid nitrogen and stored at −80˚C until further analysis. For the assessment of plasma free ^2^H-alanine enrichments, blood samples will be collected on day − 1, 0 and 10 in EDTA-containing tubes and centrifuged at 1000 g for 10 min at 4 ˚C to obtain the plasma aliquots. Additionally, saliva samples will be collected to assess body water deuterium enrichment. A background saliva sample will be collected before the first D_2_O dose on day − 1. Subsequently, saliva will be collected daily during the subsequent 10 days, in the morning before breakfast. Participants will be instructed to place a cotton swab in their mouth for approximately 1 min to saturate the cotton swab with saliva and to store the saliva samples in their freezer at home until their next visit. After the samples are collected by the research team, a syringe will be used to depress the swab to extract the saliva into a sample tube. All samples will be frozen in liquid nitrogen and stored at −80˚C until further analysis.

In the obtained muscle tissue, mixed muscle protein bound ^2^H-alanine enrichment (expressed as MPE) will be assessed using a gas chromatography-isotope ratio mass spectrometer (MAT 253; Thermo Fisher Scientific, Bremen, Germany) equipped with a pyrolysis oven using a 60-m DB-17MS column and 5-m precolumn (No. 122–4762; Agilent) and GC-Isolink. Gas-chromatography-mass spectrometry (Agilent 5975 C MSD & 7809 A GC, Wilmington DE) will be used to measure plasma free ^2^H-alanine enrichment. Body water deuterium enrichments will be assessed using gas chromatograph-combustion-isotope ratio mass spectrometry (Micromass Optima IRMS fitted with a Multiprep and Gilson auto-injector; Micromass, Manchester, UK). The analyses will be performed at the Stable Isotope Research Centre (SIRC, Maastricht University Medical Centre+ (MUMC+), Maastricht, The Netherlands) and have been described in detail previously [38].

Mixed muscle FSR will subsequently be determined using the standard precursor-product calculation [38]:

$$\mathrm{FSR}\left(\%\mathrm{day}\right)=\left(\frac{{\mathrm E}_{\mathrm m2}-{\mathrm E}_{\mathrm m1}}{{\mathrm E}_{\mathrm{precursor}}\mathrm{xt}}\right)\times100\%$$

Where *E*_m2_-*E*_m1_ represents the change in mixed muscle protein-bound ^2^H-alanine enrichments between the muscle biopsy samples taken on day 0 and day 10; *E*_precursor_ represents the plasma free ^2^H-alanine enrichment; and *t* represents the time between the biopsies taken before and at the end of the ten day measurement period.

#### Bone mineral density and bone turnover markers

Bone mineral density (BMD; g/cm^2^) will be measured at baseline and after 12 weeks using Dual X-Ray Absorptiometry (DEXA; GE Healthcare Prodigy Pro). Participants will be instructed to lie down on a table and to remain motionless during the scan. First, a total body scan will be performed to assess total body and spine BMD. Subsequently, a separate scan of each hip will be made. Additionally, blood samples will be collected at baseline, after 6 and after 12 weeks in EDTA containing tubes to assess the levels of insulin-like growth factor 1 (IGF-1), parathyroid hormone (PTH), bone resorption marker cross-linked C-telopeptide of type I collagen (CTX) and bone formation marker procollagen type I N-propeptide (PINP). Since CTX levels are highly affected by circadian rhythm, time of blood sampling will be the same for each subject and each measurement, and before 10 am [39]. The EDTA containing tube will be centrifuged for 15 min at 1000 g and 4 ˚C to obtain plasma aliquots. Plasma CTX, PINP, IGF-1 and PTH levels will be assessed using a chemiluminescent immunoassay. Plasma PTH levels will be analyzed on the same day as blood collection.

#### Metabolic profile and hs-CRP

Blood will be collected in Li-heparin and EDTA containing tubes at baseline, after 6 weeks and after 12 weeks to assess insulin levels and metabolic profile, respectively. After centrifugation at 3000 g and 22 ˚C for 8 min, plasma aliquots will be collected from the Li-Heparin tube for the assessment of insulin. The EDTA containing tube will be centrifuged for 15 min at 1000 g and 4 ˚C. Subsequently, aliquots of EDTA plasma will be collected for the determination of hs-CRP and metabolic profile. All plasma aliquots will be immediately frozen in liquid nitrogen and stored at −80˚C until further analysis. Metabolic profile will be analyzed via the commercially available high-throughput proton Hydrogen-1 Nuclear Magnetic Resonance (^1^H-NMR) metabolomics platform of Nightingale Health ltd. (Helsinki, Finland), as described elsewhere [40]. Insulin levels will be assessed using a solid-phase enzyme-labeled chemiluminescent immunometric assay.

#### Micronutrient status

Blood samples will be collected in serum, Li-Heparin, and EDTA containing tubes at baseline and after 12 weeks. The EDTA containing tube will be transported to the hospital Gelderse Vallei in Ede on the day of blood sampling for immediate analysis of hemoglobin levels using the cyanmethemoglobin method. The serum tube will be centrifuged one hour after sampling for 8 min at 3000 g and 22 ˚C. Serum aliquots will be collected for the analysis of vitamin D. The Li-Heparin tube will be centrifuged immediately after sampling for 8 min at 3000 g and 22 ˚C. Plasma aliquots will be collected for the analysis of ferritin and methylmalonic acid. All samples will be immediately frozen in liquid nitrogen and subsequently stored at −80˚C until further analysis. Vitamin D will be assessed using liquid chromatography tandem mass spectrometry. Ferritin and methylmalonic acid will be assessed using a chemiluminescent immunoassay and chromatography-tandem mass spectrometry, respectively.

#### Gastrointestinal health

Faecal samples and two aliquots of blood plasma will be collected at baseline and after 12 weeks from participants assigned to the VEG and OMNI groups. Faecal samples will be collected by the participants at home using a sterilized paper faecal bag. Subsequently, the samples are placed in sealed insulated containers by the participants, sealed in biohazard bags and stored in the freezer of the participants until their subsequent visit. Participants will be instructed to collect the faecal samples within 24 h prior to their subsequent visit to the research unit. The microbial DNA will be isolated from the faeces samples using Qiagen AllPrep DNA/RNA Mini kit (Qiagen) in combination with mechanical lysis. Metagenomic sequencing reads mapped to the human genome or aligned to Illumina adapters will be identified and removed using KneadData. The metagenomics data will reveal the taxonomy and the function of specific genes. Furthermore, blood samples will be collected in EDTA containing tubes and centrifuged at 1000 g and 4 ˚C for 15 min to obtain two aliquots of plasma. Untargeted and targeted metabolomics will be performed using LC/GC-MS on both the plasma and faecal samples. Targeted metabolomics will monitor tryptophan, tyrosine and branched-chain amino acid pathways, and oxidized amino acids. By contrast, untargeted metabolomics will explore unknown metabolic pathways and gut microbial metabolites to correlate gut microbiota functionality and circulating metabolites. By combining metagenomics with metabolomics data, and bioBakery workflow (KneadData, HUManN, MetaPhlAn, StrainPhlAN), we will investigate microbial communities using standardized and validated tools. The fusion of metagenomics and metabolomics data using Mmvec, MIMOSA2, and lineal models will link metabolites with microorganisms at strain/species level and functions.

The gastro-intestinal symptom rating scale (GSRS) questionnaire will be filled out by all participants at baseline and at the end of the intervention to assess the effect of the intervention on gastro-intestinal symptoms [41, 42]. The questionnaire includes 15 questions covering 5 common symptom clusters on a 7-point likert scale and takes approximately 3 to 5 min to fill out. These clusters are abdominal pain, reflux, indigestion, constipation and diarrhoea. Using this questionnaire, the participants will rate their symptoms over the past week.

#### Blood pressure

Systolic and diastolic blood pressure (mmHg) will be assessed at baseline, after 6 and 12 weeks. The measurement will be performed after an overnight fast using an ambulatory blood pressure monitor (Omron Healthcare Europe B.V.; OMRON automatic digital blood pressure monitor HEM-907; CE-certified). Participants will be instructed not to drink coffee, smoke or perform vigorous exercise in the 60 min before the measurement and will not be allowed to speak during the measurement. Blood pressure will be measured three consecutive times with 1 min rest intervals, after resting on a chair for 5 min. The mean of the second and third measurement will be used for analysis. A fourth measurement will be performed if the participant speaks or moves during a measurement or when a difference of 10 mmHg is observed between the second and third measurement. In those cases, the mean of the third and fourth measurement will be used for analysis.

#### Dietary assessment

Dietary intake will be assessed before the start of the intervention, at the beginning of the intervention, approximately halfway, and at the end of the intervention via three-day food records. The food records will be administered via a dietary assessment smartphone application (Traqq) [43]. The food records will cover two weekdays and one weekend day for each assessment period, which will be randomly assigned to each participant at each assessment period to allow for an even spread of dietary assessment throughout the week. Participants will receive a notification once in the morning on the day that they are assigned to report their dietary intake. After reporting of the food products, participants will be requested to enter the consumed amount and the eating occasion. The computation module of Compl-eat [44] will subsequently be used to calculate intake of food groups, energy, macro- and micronutrients by multiplying consumed amounts by energy or nutrient content using the Dutch Food Composition Database 2021, extended with the products that will be provided to the participants in this study. In case a participant has no (compatible) smartphone, the participant will list his or her dietary intake on a paper food diary, which will subsequently be transferred to Compl-eat by a member of the research team. Participants will be contacted for clarifications in case of obscurities in the dietary data. All records will be checked by a member of the research team for completeness, unusual consumed amounts, and notes.

#### Assessment of physical activity

Participants will wear an accelerometer (ActiGraph, Pensacola, Florida; CE: EN60601-1) on their hip during seven consecutive days before the start of the intervention, during the first 10 days of the intervention and during seven consecutive days in the last month of the intervention to assess physical activity levels. Additionally, participants will be instructed to record their activity in a diary during the days that the accelerometer is worn.

#### Other study outcomes

Baseline participant characteristics will be collected during screening and at baseline using questionnaires. Baseline characteristics include demographics (gender, age, ethnicity, living situation, education, smoking habits, alcohol use), medical history, lifestyle habits, body mass (kg), height (m), and medication and supplement use. Body mass will be measured to the nearest 0.1 kg during screening, at baseline, after 6 weeks and after 12 weeks using a calibrated digital scale. Height will be measured to the nearest 0.01 m during screening using a wall mounted stadiometer. Handgrip strength (kg) will be measured at baseline with the use of a hand dynamometer (Jamar, Jackson, Michigan). Both hands will be measured three consecutive times to the nearest 0.5 kg. During the measurement, participants will be seated in an upright position with the arm in a 90-degree angle. Furthermore, whole blood samples will be collected in Li-Heparin tubes at baseline and week 12 to assess DNA methylation. DNA methylation will be assessed using standard bisulfite methods and assays like Illumina 450 K, EPIC 850 K, or largely similar methods. In support of the DNA methylation assessment, whole blood samples will be collected in EDTA containing tubes and analyzed to assess the differentiation of leukocytes (basophils, eosinophils, segmented leukocytes, lymphocytes, and monocytes) on the same day of blood collection using hemocytometry. Additionally, participants that report to have (osteo)arthritis at the moment of screening, will be asked to fill out the Western Ontario and McMaster Universities Arthritis Index (WOMAC) at baseline and after 12 weeks [45, 46].

#### Adverse events

Adverse events will be recorded in Castor EDC [31] and checked regularly by the research physician. The accredited medical ethical committee will be notified in the case of serious adverse events. All adverse events will be followed up until they have abated or until a stable situation has been reached.

### Statistical analysis plan

All data will be treated according to the General Data Protection Regulation Act (EU GDPR) and will be collected using Castor EDC [31]. Analyses will be performed in R version 4.1.0. Prior to the analysis, group allocation will be blinded to the members of the research team that will perform the statistical analysis. Descriptive statistics will be used to explore and present baseline characteristics of the study population. Continuous data will be presented quantitatively as means and their 95% confidence intervals or, when not normally distributed, as median [interquartile range]. Categorical data will be presented as counts and percentages. Data will be analysed according to the intention-to-treat principle.

Independent samples t-tests will be performed to analyse the difference in change of thigh muscle volume and the difference in mixed MPS between groups VEG and OMNI and between groups VEG and VEG-RE. Data will be checked for normality using QQ-plots. Missing data may be imputed via multiple imputation or the related data may be eliminated. In case of a non-normal distribution, non-parametric tests will be used or data will be log-transformed. Furthermore, linear mixed models will be used to assess the differences between these groups for the other outcome measures. Participants will be added to the model as random factors. Treatment and timepoint will be added as fixed factors as main effects and their interaction. A maximum of three potential confounding factors may be added to each model as fixed factor. Model assumptions will be checked using QQ-plots and scatterplots. A two-sided p-value < 0.05 will be considered statistically significant. The Benjamini-Hochberg correction will be applied to adjust for multiple testing. In case more than two time points are tested and a significant treatment*time interaction is observed, a post-hoc analysis for will be performed using a Bonferroni correction.

## Discussion

The main aims of the Vold-study are to assess the effects of a self-composed vegan diet in comparison to an omnivorous diet on thigh muscle volume in healthy, community-dwelling, older individuals, and to investigate whether RE may attenuate any potential reductions in muscle volume while consuming a vegan diet. To our knowledge, this is the first trial to assess the health effects of a longer term self-composed vegan diet in older adults. The high environmental impact of animal-based food production and health benefits related to the increased consumption of plant-based foods have led to the adoption of more plant-centered dietary guidelines [1–3], and the increased implementation of plant-based meals and diets by consumers and healthcare institutions [47–49]. However, the consequences of longer term adherence to a self-composed fully plant-based, i.e. vegan, diet for skeletal muscle and other health outcomes in older individuals remain unknown.

Previous studies have assessed the acute effects of the consumption of plant-based protein isolates [20, 50, 51], a vegan meal [26] and a vegan diet [23, 24] on MPS in older individuals in highly controlled settings. Monteyne et al. [24] observed no difference in MPS rates during a three day controlled mycoprotein based vegan diet with a high protein content in comparison to an isonitrogenous omnivorous diet. Additionally, we recently observed no differences in MPS between a highly controlled vegan and omnivorous diet with ample protein in older adults as well [23]. Nevertheless, we expect that a self-composed, longer term, vegan diet will differently impact skeletal muscle in our older participants during the 12-week intervention period. Although highly controlled dietary intervention studies are essential in assessing the inherent differences between specific components of a food product or diet, longer term adherence to a self-composed diet will result in the consumption of different food products, and hence nutrients. The framework of our intervention was therefore specifically chosen to allow for a higher external, especially ecological, validity of the results that will be obtained [52].

The potentially lower anabolic properties of a vegan diet poses a risk for the skeletal muscle health of older individuals, while a vegan diet may concurrently improve risk factors of other age-related non-communicable diseases, as cardiometabolic risk factors [4–6]. By assessing the effects of the intervention on a wide range of other health outcomes as well (Table 1), we anticipate that the results of the Vold-study will provide an extensive overview regarding the consequences of shifting to a vegan diet at an older age, considering both the potential risks and benefits of such a shift. Additionally, although no major nutrient insufficiencies are expected to transpire due to the relatively short duration of the intervention, assessing the status of vitamin D, methylmalonic acid, ferritin and haemoglobin at baseline and at the end of the intervention will provide valuable insights into the risk of development of insufficiencies of cobalamin, vitamin D and iron while consuming a vegan diet later in life. The Vold-study is the first trial to assess the effects of a self-composed vegan diet later in life on such an extensive range of health outcomes. The results will therefore contribute to the substantiation of the envisaged more plant-based dietary guidelines [3, 53].

Given the extent and complexity of the presently proposed study, several challenges and potential limitations will have to be considered. First, the high number of measurements, complexity of the intervention, and the duration of the trial may complicate the recruitment of ample participants. We therefore plan to set out a broad range of recruitment strategies, ranging from (commercial) volunteer databases to advertisements in online and offline media. Second, shifting to a vegan diet is complex and our participants will require adequate guidance. However, as the aim of the Vold-study is to assess the consequences of a *self-composed* vegan diet, it will be essential not to steer participants too much in their dietary choices either. By informing our participants during monthly meetings guided by trained dietitians and by providing practical tools, as pocket-sized hand-outs with (non)vegan ingredients, product logo’s, recipes, product lists, and bi-weekly provision of plant-based food products, we envisage to adequately guide our participants through their shift to a vegan diet, while not imposing our participants with strict rules. Third, we are aware of the possibility that the control group may adopt a more plant-based diet due to increased adherence to the national dietary guidelines and because participants may already have a specific interest in plant-based diets when registering for the Vold-study. We will anticipate on this by providing the participants in the control group with animal-based food products every other week. Additionally, by including participants that habitually consume animal-based food products on at least five days per week and instructing them not to lower their animal-based food intake, we expect no large reductions in animal-based food consumption in the control group. Fourth, maintaining high compliance is a well-known challenge in dietary intervention studies [54]. We aim to keep adherence high by providing self-selected food-products bi-weekly to all participants, both assisting them in adhering to their allocated group as well as allowing us to have bi-weekly contact moments with all our participants. Furthermore, the social aspects of the group meetings, exercise sessions and the possibility of trying new food products will be highlighted throughout the intervention period. Last, participant dropout may result in missing data. To prevent any problems related to participant dropout, we accounted for 20% dropout in our sample size calculation. We further envisage that the aforementioned strategies to improve compliance, will reduce the number of dropouts in the Vold-study. Additionally, participants that withdraw before the initiation of the intervention diets will be replaced. To allow for intention to treat analysis, dropouts will be asked, but not obliged, to visit the research facilities for follow-up measurements.

Although complex, the design of the Vold-study encompasses the important strength of using several gold standard methodologies, as MRI-scanning, the Biodex measurement, and DEXA-scanning for the assessment of muscle volume, fat distribution, muscle strength and bone mineral density, respectively. Magnetic resonance imaging is considered the gold standard in assessing muscle volume as well as fat distribution [55, 56], and its sensitivity will allow us to pick up small changes over the 12-week intervention period. As such, we envisage that we are able to observe the expected changes within the relatively short timeframe of 12-weeks, whereas a longer study duration would have been needed if less a sensitive measurement method was chosen. The same applies for the Biodex measurement, which is a widely used method to accurately assess muscle strength [57, 58]. Using these high standard methods to assess muscle volume, MFI as well as muscle strength, enables us to gain valuable insights in overall change in muscle quality and function in response to the intervention rather than in muscle volume alone. Loss of muscle strength and function are, in addition to muscle mass, strong predictors for adverse health outcomes and are key characteristics in the identification of sarcopenia [59]. Furthermore, additionally assessing the short-term effects on mixed MPS rates, allows us to investigate to what extent the differences between groups in mixed MPS response translate to the longer term differences in muscle volume. Although the effects of RE on myofibrillar MPS rates correlate well with training induced muscle hypertrophy in trained individuals, it remains to be explored to what extent the acute effects of a dietary intervention on mixed MPS rates translate into longer term adaptations in thigh muscle volume [60, 61].

This 12-week randomized controlled trial aims to assess the effects of a self-composed vegan diet, with or without coinciding RE, on muscle related outcomes and a broad range of other health related outcomes in older adults. The findings will fill major gaps in the literature regarding the health consequences of shifting to a vegan diet later in life, and will provide insights regarding the health implications of the envisaged shift towards more plant-based dietary guidelines.

## Supplementary Information

Supplementary Material 1.

## Data Availability

No datasets were generated or analysed during the current study.

## Abbreviations

ASAT: Abdominal subcutaneous adipose tissue
BMD: Bone mineral density
BMI: Body mass index
CTX: C-telopeptide of type I collagen
COPD: Chronic obstructive lung disease
D_2_O: Deuterium oxide
DEXA: Dual X-ray absorptiometry
FSR: Fractional synthesis rates
GDPR: General Data Protection Regulation Act
g/kg/d: Grams per kilogram per day
GSRS: Gastro-intestinal Symptom Rating Scale
hs-CRP: High sensitive – C-reactive protein
IGF-1: Insulin-like growth factor 1
LFF: Liver fat fraction
MFI: Muscle fat infiltration
MPE: Mole percent excess
MPS: Muscle protein synthesis
MRI: Magnetic resonance imaging
MVC: Maximal voluntary contraction
OMNI: Group following their habitual omnivorous diet
P1NP: Procollagen type I N-terminal propeptide
PTH: Parathyroid hormone
RE: Resistance exercise
RM: Repetition maximum
TE: Echo time
TR: Repetition time
VAT: Visceral Adipose tissue
VEG: Intervention group allocated to follow a vegan diet
VEG-RE: Intervention group allocated to follow a vegan diet and RE
WOMAC: Western Ontario and McMaster Universities Osteoarthritis Index
